# Docosapentaenoic acid and lung cancer risk: A Mendelian randomization study

**DOI:** 10.1002/cam4.2018

**Published:** 2019-02-11

**Authors:** Jiaqing Liu, Huaqiang Zhou, Yaxiong Zhang, Yan Huang, Wenfeng Fang, Yunpeng Yang, Shaodong Hong, Gang Chen, Shen Zhao, Xi Chen, Zhonghan Zhang, Jiayi Shen, Wei Xian, Jianhua Zhan, Yuanyuan Zhao, Xue Hou, Yuxiang Ma, Ting Zhou, Hongyun Zhao, Li Zhang

**Affiliations:** ^1^ Department of Medical Oncology Sun Yat‐sen University Cancer Center Guangzhou China; ^2^ State Key Laboratory of Oncology in South China Guangzhou China; ^3^ Collaborative Innovation Center for Cancer Medicine Guangzhou China; ^4^ Zhongshan School of Medicine Sun Yat‐sen University Guangzhou China

**Keywords:** Causality, Docosapentaenoic acid, Lung cancer, Mendelian randomization, Polyunsaturated fatty acid

## Abstract

**Background:**

Observational studies have shown that excessive dietary fat may be associated with lung carcinogenesis. However, findings from previous studies are inconsistent and it remains unclear whether docosapentaenoic acid (DPA), a kind of polyunsaturated fatty acid, is linked to the risk of lung cancer. The aim of this study is to investigate the causal effect of DPA on lung cancer with Mendelian randomization (MR) method.

**Methods:**

With a two‐sample MR approach, we analyzed the summary data from the Cohorts for Heart and Aging Research in Genomic Epidemiology (CHARGE, 8866 individuals of European ancestry) Consortium and International Lung Cancer Consortium (ILCCO, 11 348 lung cancer cases and 15 861 controls; European ancestry) to assess the possible causal relationship of DPA on the risk of lung cancer.

**Results:**

Our results indicated that genetically predicted higher DPA level has a positive association with lung cancer, where 1% higher DPA was associated with a 2.01‐fold risk of lung cancer (odds ratio [OR]: 2.01, 95% CI = 1.34‐3.01; *P* = 7.40 × 10^−4^). Additionally, lung cancer was not a causal factor for DPA. The results of MR‐Egger regression analysis showed that there was no evidence for the presence of directional horizontal pleiotropy.

**Conclusions:**

Genetically elevated DPA is positively associated with risk of lung cancer, and more work is needed to investigate the potential mechanisms.

## 1 INTRODUCTION

Lung cancer is one of the most common cancers in the world, with an estimated 234 030 new cases in 2018.^1^ According to a systemic analysis for the global burden of disease study, lung cancer was the leading cause of cancer deaths and Disability Adjusted Life Years (DALYs), with 1.2 million deaths and 25.4 million DALYs.^2^ Advances in early detection of lung cancer and management of cancer patients can help to reduce the burden of lung cancer. Additionally, it is vital to identify the modifiable and avoidable risk factors for primary prevention, which can significantly lower the risk of cancer by preventing exposures to hazards, altering risky behaviors. For example, smoking is seen as the number one risk factor, which is linked to about 80%‐90% of lung cancers.^3^, ^4^, ^5^ Effective smoking cessation has played a crucial role in reducing the incidence of lung cancer as well as improving the survival of the patients.^6^, ^7^, ^8^ Lung cancer in never‐smokers, nevertheless, is an increasingly prominent public health issue. An estimated 10%‐15% of all lung cancers are attributed to factors other than tobacco, and lung cancer in never‐smokers causes 16 000‐24 000 deaths in America annually.^9^, ^10^ Therefore, research is still required exploring other potentially modifiable risk factors in order to further reduce the lung cancer burden.

Many prevention practices have indicated that proven causal relationship between dietary factors and cancer is the basis of dietary guidelines, which can provide recommendations for cancer prevention.^11^ Polyunsaturated fatty acids (PUFAs) are one kind of important nutrients related to carcinogenesis and potential anticancer effect.^12^ Among them, N3 polyunsaturated fatty acids (n3 PUFAs) have been associated with prevention in cancers such as colon cancer, prostate cancer, and breast cancer.^13^ In the past, many studies mainly gave priority to the effect of docosahexaenoic acid (DHA) and eicosapentaenoic acid (EPA) and found that they could serve as latent cytotoxic therapeutic role against lung cancer.^14^, ^15^, ^16^, ^17^ Both DHA and EPA could inhibit the proliferation of the human lung adenocarcinoma cell line A549 and induce cell apoptosis and autophagy.^18^ Currently, many studies began to provide evidence to illustrate the significant biological effect of docosapentaenoic acid (DPA) separately, a kind of PUFA which shares many structural similarities with EPA and DHA.^19^, ^20^ The anti‐proliferative effect of DPA has been reported in colorectal carcinoma.^21^ However, due to the high cost and the difficulty of the purification of DPA, previous studies on DPA were still very limited.^19^ The association between DPA and lung cancer has not been systematically examined. Whether there is a causal relationship between DPA and lung cancer remains unknown.

Mendelian randomization (MR) is a new approach that could provide evidence about the putative causal relationship between modifiable risk factors and disease.^22^, ^23^ It is based on the random allocation of alleles at conception and the independent assortment of genes for different traits, by using several genetic instruments as a proxy for exposure. With genetic variants used as instrumental variables for risk factors, MR can be regarded as a natural analogue of classical randomized controlled trials (RCTs), which could find out whether the risk factors are causal for the disease conveniently.^23^ Compared to classical RCTs, MR also has advantages such as being time‐saving, cost‐effective, and feasible. Besides, reverse causality, which leads to bias in conventional observational studies, is avoided because the process between gene and disease is usually a unidirectional flow.^24^ MR method has been successfully applied in several studies about the causality between PUFAs and risk of cancers, such as prostate cancer, colorectal cancer, and melanoma.^25^, ^26^, ^27^ But it still remains blank about DPA in the field of lung cancer.

In this study, we aimed to identify a potentially causal association between DPA and risk of lung cancer using an MR analysis.

## 2 MATERIAL AND METHODS

### 2.1 GWAS summary data

The main analysis used publicly available genetic summary data from two large consortiums Cohorts for Heart and Aging Research in Genomic Epidemiology Consortium (CHARGE) and International Lung Cancer Consortium (ILCCO).^28^, ^29^ CHARGE and ILCCO consortium have kindly made their summary data available in the MR‐Base platform, which is a great platform that supports MR analysis.^30^ Thus, genome‐wide association studies (GWAS) summary data used in this study are publicly available without the need for application through the MR‐Base platform, which is accessible at http://www.mrbase.org/.

### 2.2 Genetic variants associated with DPA

We mainly used the publicly available GWAS summary data from CHARGE Consortium. Lemaitre et al identified three single nucleotide polymorphisms (SNPs) (rs780094, rs3734398, rs174547) robustly associated with plasma DPA levels at a GWAS threshold of statistical significance (*P* < 5*10^‐8^; linkage disequilibrium *r* ^2^ < 0.1). For each SNP selected, the summary data (the effects of each of SNPs on DPA; effect sizes and standard errors) were derived from published GWAS conducted by the CHARGE consortium through MR‐Base platform (8866 individuals of European ancestry).^28^, ^30^ Finally, we utilized these three independent SNPs as instrumental variables that associated with plasma DPA levels.^25^, ^26^ Our selection of SNPs as instrumental variables for DPA is consistent with previous studies by Khankari et al and May‐Wilson et al.^25^, ^26^

### 2.3 Genetic variants associated with lung cancer

Publicly available GWAS summary data on lung cancer were retrieved from ILCCO consortium (11 348 lung cancer cases and 15 861 controls; European ancestry).^29^ For each of the three SNPs associated with DPA (rs780094, rs3734398, rs174547), we retrieved summary data (the effects of each of SNPs on lung cancer, effect sizes, and standard errors) for the same SNPs through MR‐Base platform.

### 2.4 Statistical analyses

We adopted a strategy called two‐sample MR to perform our analysis, which allows us to analyze without individual patient data.^31^ All analyses were conducted in R (version 3.4.2) with the package “TwoSampleMR” (version 0.3.4). With the SNP‐exposure effects and the SNP‐outcome effects obtained from different studies, we estimated the causal influence of DPA on lung cancer risk and harnessed the statistical power of pre‐existing GWAS analyses. Our two‐sample MR analysis utilized summary data from two different studies, CHARGE and ILCCO consortium.^28^, ^29^ Both studies were comprised of populations of European ancestry. For all Mendelian randomization analyses, alleles from the CHARGE and ILCCO datasets, were aligned to correspond to an increase in DPA. We used three Mendelian randomization approaches to determine MR estimates of DPA for lung cancer (inverse‐variance weighted [IVW] approach, weighted median method, and MR‐Egger method). First, we conducted a random‐effects IVW meta‐analysis approach, by regressing the SNP‐DPA associations against the SNP‐Lung cancer associations and calculating the inverse variance weighted mean of ratio estimates from three instruments (rs780094, rs3734398, rs174547).^31^ We conducted the random‐effects IVW instead of a fixed‐effects IVW, because the fixed‐effects IVW was restricted to the assumption that none of the selected SNPs exhibit horizontal pleiotropy, while random‐effects IVW allowed each SNP to have different mean effects.^32^ Second, we estimated the effects using weighted median methods. This approach helped us figure out the weighted empirical distribution function of ratio estimates of all the selected SNPs. Only 50% of the SNPs need to be valid instruments to ensure that the causal effect estimate would be unbiased, since the weighted median estimate allows SNPs with stronger effect to contribute more toward the estimate.^33^ Third, we conducted a MR‐Egger analysis. This method assumes that the horizontal pleiotropy are not associated with the SNP‐exposure effects, known as InSIDE (Instrument Strength Independent of Direct Effect) assumption,^34^ allowing a non‐zero intercept in the regression and unbalanced horizontal pleiotropy across all SNPs. The MR‐Egger regression is a weighted linear regression of SNP‐lung cancer risk against SNP‐DPA effect estimates. It could provide valid effect estimate even if all SNPs are invalid instruments. Additionally, we performed the same analysis for different histologic subtypes (adenocarcinoma (LUAD) and squamous cell carcinoma (LUSC)). Results are available in odds ratio (OR) and 95% confidence interval (CI), which provide an estimate of relative risk caused by percentage of DPA levels per total plasma fatty acids.

There are three instrumental variable assumptions for MR method: the instrumental variables are strongly associated with DPA; the instrumental variables affect lung cancer only through their effect on DPA; and instrumental variables are independent of any confounders of the association between DPA and lung cancer.^35^ Therefore, we performed a MR‐Egger sensitivity test to measure the degree of directional horizontal pleiotropic effects might bias the Mendelian randomization causal estimates.^36^ We also performed a leave‐one‐out analysis to assess whether the MR estimate is driven or biased by a single SNP.

To investigate whether lung cancer might be a causal factor for DPA, we performed a Mendelian randomization analysis in the opposite direction using three SNPs linked to lung cancer.

## 3 RESULTS

### 3.1 Causal effect from DPA to lung cancer

**Figure 1:**
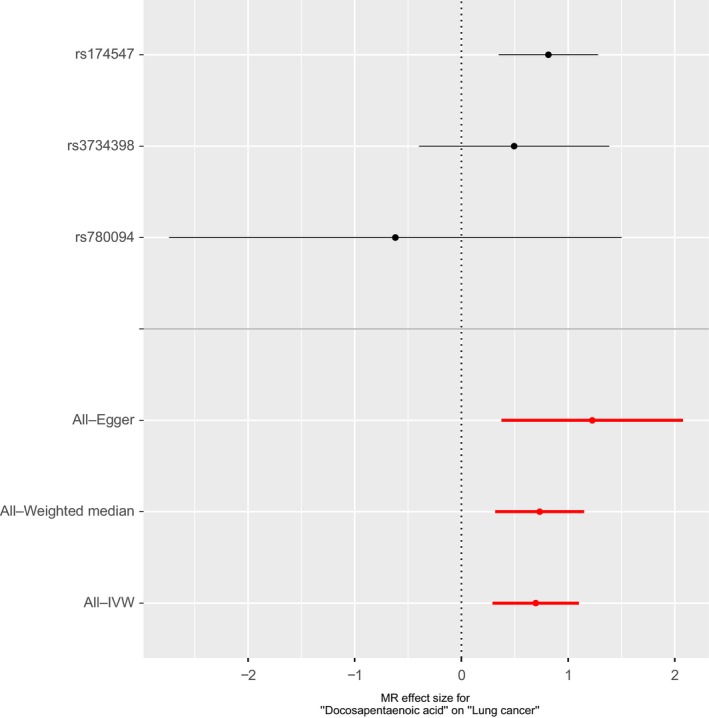
Forest plot of single nucleotide polymorphisms (SNPs) associated with docosapentaenoic acid (DPA) and their risk of lung cancer. The forest plot shows association of genetic liability to DPA level on lung cancer. Each black point represents the log odds ratio (OR) for lung cancer per standard deviation (SD) increase in DPA, produced using each of the DPA SNPs (rs174547, rs3734398, rs780094) as separate instruments. Red points show the combined causal estimate using all SNPs together in a single instrument, with three different methods (inverse‐variance weighted [IVW] approach, MR‐Egger, and weighted median). Horizontal line segments denote 95% confidence intervals of the estimate

**Figure 2:**
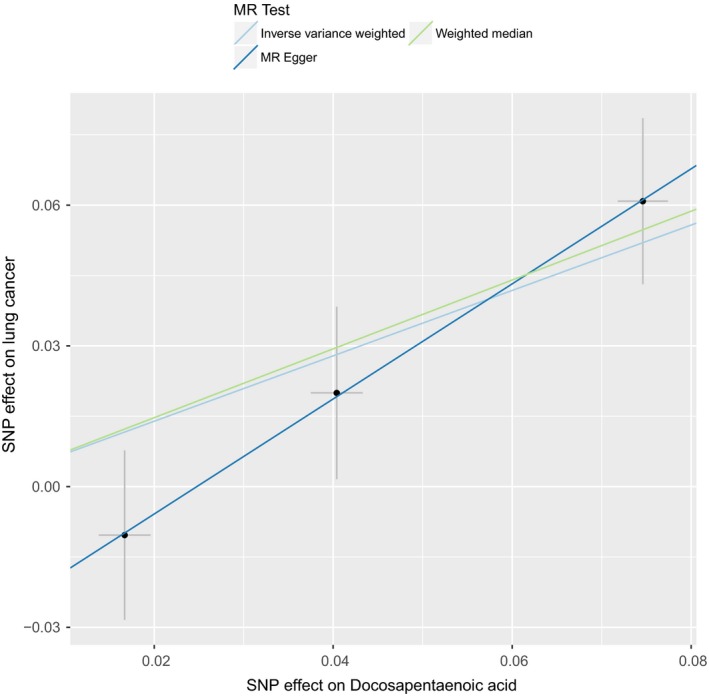
Scatter plot of SNPs associated with DPA and their risk of lung cancer**.** A plot relating the SNP effect on DPA (x‐axis, SD units) and SNP effect on lung cancer (y‐axis, log(OR)) with 95% confidence intervals. The Mendelian randomization (MR) regression slopes of the lines correspond to the causal estimates using each of the three different methods (IVW approach, MR‐Egger, and weighted median). The light blue line shows causal regression estimates from IVW. The deep blue line shows causal regression estimates from MR‐Egger. The green line shows causal regression estimates from weighted median

To investigate the causal effect from DPA to lung cancer, we conducted the conventional Mendelian randomization analysis (IVW method). Our two‐sample MR analysis indicated that, genetically predicted higher DPA level was associated with significantly higher risk of lung cancer Table 1. One per cent higher DPA was associated with a 2.01‐fold risk of lung cancer (OR 2.01, 95% CI = 1.34‐3.01, *P* = 7.40 × 10^−4^). The causal estimates were similar in terms of direction and magnitude using MR‐Egger and weighted median method Table 1. Figure 1 showed individual causal estimates from each of the three SNPs (rs780094, rs3734398, rs174547), among which rs174547 showed significant effect on the association between DPA and lung cancer. The combined causal effect of all the three SNPs was also depicted in Figure 1, with three different methods. That means associations were consistent in analyses using different methods. The Mendelian randomization regression slopes were illustrated in Figure 2.

**Table 1:** Mendelian randomization estimates of the associations between docosapentaenoic acid and risk of lung cancer overall and histologic types

Outcome	IVW method		MR‐Egger		Weighted median method	
	OR (95% CI)	*P* value	OR (95% CI)	*P* value	OR (95% CI)	*P* value
Lung cancer overall	2.01 (1.34‐3.01)	7.40e‐04*	3.41 (1.46‐7.98)	0.22	2.08 (1.37‐3.16)	5.49e‐04*
Adenocarcinoma	2.54 (1.38‐4.69)	2.84e‐03*	3.77 (1.03‐13.85)	0.30	2.58 (1.38‐4.82)	2.88e‐03*
Squamous cell carcinoma	2.20 (1.18‐4.10)	1.29e‐02*	4.21 (1.15‐15.39)	0.27	2.36 (1.24‐4.50)	9.11e‐03*

IVW, inverse‐variance weighted; OR, odds ratio; CI, confidence interval.

* *P* value < 0.05.

**Figure 3:**
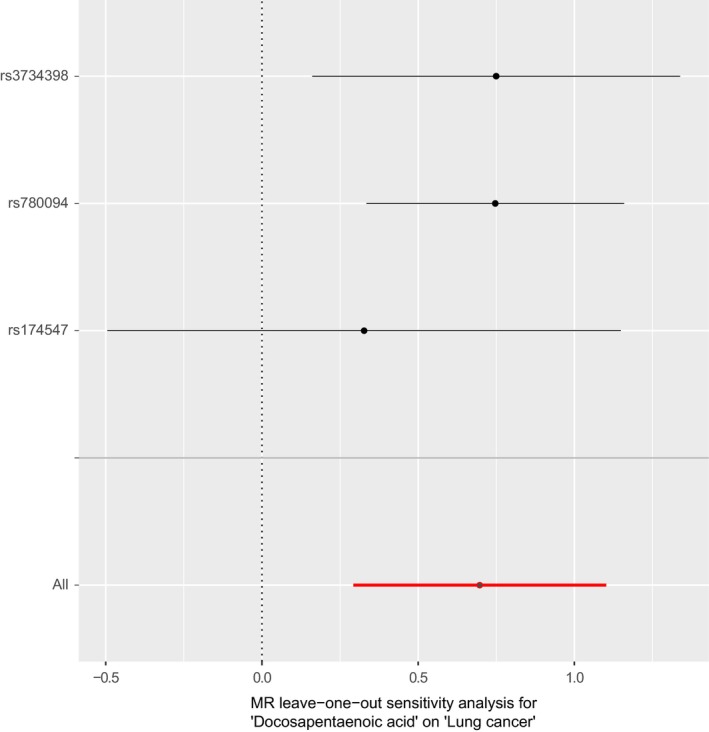
Leave‐one‐out of SNPs associated with DPA and their risk of lung cancer. Each black point depicts the causal estimate of DPA on lung cancer excluding particular SNP (rs3734398, rs780094, and rs174547, respectively) from the analysis. The red point depicts the IVW estimate using all SNPs. The leave‐one‐out analysis shows no single SNP was strongly or reversely driving the overall effect of DPA on lung cancer. Rs174547 plays a relatively predominant role in the association between DPA and lung cancer according to the leave‐one‐out analysis

In a leave‐one‐out sensitivity analysis, no single SNP was strongly or reversely driving the overall effect of DPA on lung cancer Figure 3. Rs174547 seems to play a relatively predominant role in the association between DPA and lung cancer according to the leave‐one‐out analysis. Considering that rs174547 is related to serum DPA level as well as many other metabolites, including ALA, EPA, ARA, DHA, etc, we further perform additional Mendelian randomization analysis to explore the relationship between these metabolites related to rs174547 and lung cancer. We found that DHA (*P* = 0.44, IVW method), EPA (*P* = 0.14, IVW method), ARA (*P* = 0.41, IVW method) were not associated with lung cancer risk, and that ALA protected against lung cancer (OR 0.02, 95% CI = 0.00‐0.19, *P* = 5.9 × 10^‐4^, IVW method). Besides, rs174547 and rs174548 are in high linkage disequilibrium. According to the findings of Wang et al, rs174548 is related to lung cancer risk (ORmeta = 0.87, 95% CI = 0.84‐0.90, *P*meta = 1.76 × 10^‐15^).^37^ Our further analysis also showed that, when taking rs174548 as a single instrumental variable, DPA was associated with lung cancer risk (OR = 2.42, 95% CI=1.54‐4.01, *P* = 2.02 × 10^‐4^), which was similar to that of rs174547 (OR = 2.26, 95% CI = 1.42‐3.60, *P* = 5.90 × 10^‐4^). To some extent, these results indicated the heterogeneous effect of PUFAs on lung cancer, and implied that DPA as the potential mechanism of increased risk in lung cancer. There was no evidence for the presence of directional horizontal pleiotropy in the MR‐Egger regression analysis Table 2. *P* values for the intercept were large and the estimates adjusted for pleiotropy suggested null effects (intercept *β* = −0.03, *P* = 0.40). The similar causal trend was observed in both LUAD and LUSC (LUAD, OR 2.54, 95% CI = 1.38‐4.69, *P* = 2.84 × 10^−3^, LUSC, OR 2.20, 95% CI = 1.18‐4.10, *P* = 1.29 × 10^−2^) Table 1, Figures S1‐S6.

**Table 2:** MR‐Egger pleiotropy test of the associations between docosapentaenoic acid and risk of lung cancer overall and histologic types

Outcome	MR‐Egger method	
	Intercept	*P* value
Lung cancer overall	−0.03	0.40
Adenocarcinoma	−0.02	0.62
Squamous cell carcinoma	−0.04	0.47

MR, Mendelian randomization.

### 3.2 Causal effect from lung cancer to DPA

Lung cancer was not causally associated with DPA levels (OR 1.02, 95% CI = 1.00‐1.03, *P* = 0.05). Causal effect was consistent after application of other statistical methods, such as MR‐Egger and weighted median methods Table 3. Additional MR routine results were shown in Figures S7‐S9.

**Table 3:** Mendelian randomization estimates of the associations between lung cancer and docosapentaenoic acid

Methods	OR (95% CI)	*P* value
IVW	1.02 (1.00‐1.03)	0.05
MR‐Egger	0.99 (0.88‐1.11)	0.89
Weighted median	1.01 (1.00‐1.03)	0.16

IVW, inverse‐variance weighted; OR, odds ratio; CI, confidence interval.

## 4 DISCUSSION

Much work has focused on modifiable risk factors that have potential causal relationship with cancer. Dietary factors are attached with great importance, because proven causal relationship between diet and cancer can help public health policymakers to develop dietary guidelines, which provide recommendations for cancer prevention.^11^ Among them, dietary PUFAs are one type of important nutrients that are associated with cancers.^12^ In this large Mendelian randomization study, we examined a potential causal effect of DPA on lung cancer, using GWAS summary data obtained from two large consortiums. Our results suggested that a 1% increase in DPA levels was associated with roughly twofold risk of lung cancer overall regardless of different histology. In contrast, lung cancer does not contribute to higher DPA levels.

The results of previous studies on the association between PUFAs and lung cancer were inconclusive. Evidence from studies in animal models and human lung cancer cell has shown both DHA and EPA could inhibit the progression of NSCLC.^18^, ^38^, ^39^ The prospective study conducted by Luu et al revealed that total PUFAs intake could lower the risk of lung cancer while DHA or EPA intake increased risk of lung cancer in female never‐smokers.^40^ However, a systematic review and meta‐analysis conducted recently indicated that fish consumption, which provides rich DHA and EPA, was associated with a decreased risk of lung cancer.^41^ Meanwhile, another meta‐analysis based on eight prospective cohort studies demonstrated that PUFA intake had no significant influence on lung cancer risk and might be beneficial for female in lung cancer prevention.^42^ Although the important biological effects of DPA are supported by a lot of evidence, no study has specially evaluated DPA for its association with lung cancer risk, which might result from the fact that pure DPA has not been available at an affordable price.^19^ Highly purified DPA has been difficult to isolate and the purification costs a lot, thus making large‐scale intervention studies in humans not possible at the moment.^43^, ^44^ Due to the restriction, conventional observational studies have not addressed clear guidance on the role of DPA in lung cancer risk. Hence, we explored the causal association of DPA with lung cancer using an MR approach, which is cost‐effective and fast.

To the best of our knowledge, our work is the first study to appraise the causality between DPA and lung cancer risk. Based on previous research results, DPA has been linked to better health and lower total mortality.^45^, ^46^, ^47^ The possible mechanisms of the protective effect of DPA include inhibiting platelet aggregation, stimulating endothelial cell migration, reducing age‐related oxidative damage, and inhibiting inflammation.^48^, ^49^, ^50^, ^51^ But interestingly, our analysis revealed that DPA intake was associated with an increased risk of lung cancer in European people unexpectedly, which was contrary to lots of observational studies. It suggested that the potential adverse effect of DPA on lung cancer patients should be considered when public health policymakers develop dietary guidelines on lung cancer prevention.

Pure DPA has been difficult to isolate and not readily available yet; hence, the mechanisms that mediate the cause‐effect relationship between DPA and lung cancer have not hitherto been studied and reported. Limited literature on the adverse effect of DPA is therefore difficult to figure out the potential mechanisms to interpret our finding now. As a kind of PUFA with active biological effect, DPA is prone to become a greater focus of research in the area of lung cancer. More work is needed to explore the molecular mechanism in the future. Furthermore, previous study revealed that the ratio between n‐6 PUFAs and n‐3 PUFAs was inversely associated with lung cancer risk.^40^ As a whole, n‐3 PUFAs is a kind of important nutrient that contributes to human development, health, and well‐being.^52^ Thus, maybe it is important to figure out what ratio between DPA and other n‐3 PUFAs as well as n‐6 PUFAs benefits human health most.

Our analysis presents several important strengths. First, the MR design can prevent reverse causation and potential confounding factors that are generally present in conventional observational studies. Second, we used a two‐sample MR approach to analyze the summary data generated from two different studies, which has an advantage over one sample MR. The effect estimates are more accurate than that from a single study because statistical power increases as the sample size becomes larger.^53^ There is one more point, it would be costly and time‐consuming to conduct a classical RCT with purified DPA, which is intensely difficult to implement now. In contrast, our study is cost‐effective, economical with time and effort.

However, there are few limitations to our study. First, the data that support the findings of this study were collected from two large consortiums that were of European origin. The generalizability of our findings needs to be confirmed. Second, our results were based on analysis of the GWAS summary data, which can result in overestimation of the SNP‐trait effect, because SNP with the smallest *P* value was usually selected as the lead SNP in the GWAS report and the associations for other significant SNPs were not reported, also known as Beavis effect.^54^ That is to say, our study might overestimate the association between DPA and the risk of lung cancer because of the potential association between DPA and confounders in the GWAS discovery stage. Third, we did not have access to individual patient data of the cohort studied, thus making it impossible for us to conduct subgroup analysis to include covariates in our study. In addition, the underlying biology or mechanisms of the association between DPA and lung cancer is totally unknown yet and we cannot explore the probable mechanism through MR methods. Therefore, our findings might be counterintuitive and were required to be confirmed by further studies since the previous studies have depicted that DPA might be beneficial for health.

## 5 CONCLUSIONS

In conclusion, using data from two large consortiums, we report an overall positive association between DPA and lung cancer risk. More work is needed to investigate the potential mechanisms and elucidate the roles of DPA in the etiology of lung cancer clearly.

## CONFLICT OF INTEREST

All authors have no conflicts of interested to declare.
