# Supplementary material for: Docosapentaenoic acid and lung cancer risk: A Mendelian randomization study
Source: Cancer Med. 2019 Feb 11;8(4):1817–25. doi: 10.1002/cam4.2018 (PMC6488117; doi:10.1002/cam4.2018)
Supplement: Supplementary file 1 [file CAM4-8-1817-s001.pdf]

Docosapentaenoic Acid and Lung Cancer Risk: a Mendelian Randomisation Study (Supplementary Materials)

Contents:

Supplementary Figures

Fig. S1. Forest plot of single nucleotide polymorphisms (SNPs) associated with DPA and their risk of lung adenocarcinoma.

Fig. S2. Scatter plot of SNPs associated with DPA and their risk of lung adenocarcinoma.

Fig. S3. Leave-one-out of SNPs associated with DPA and their risk of lung adenocarcinoma.

Fig. S4. Forest plot of SNPs associated with DPA and their risk of squamous cell lung cancer.

Fig. S5. Scatter plot of SNPs associated with DPA and their risk of squamous cell lung cancer.

Fig. S6. Leave-one-out of SNPs associated with DPA and their risk of squamous cell lung cancer.

Fig. S7. Forest plot of SNPs associated with lung cancer and their risk of DPA.

Fig. S8. Scatter plot of SNPs associated with lung cancer and their risk of DPA.

Fig. S9. Leave-one-out of SNPs associated with lung cancer and their risk of DPA.

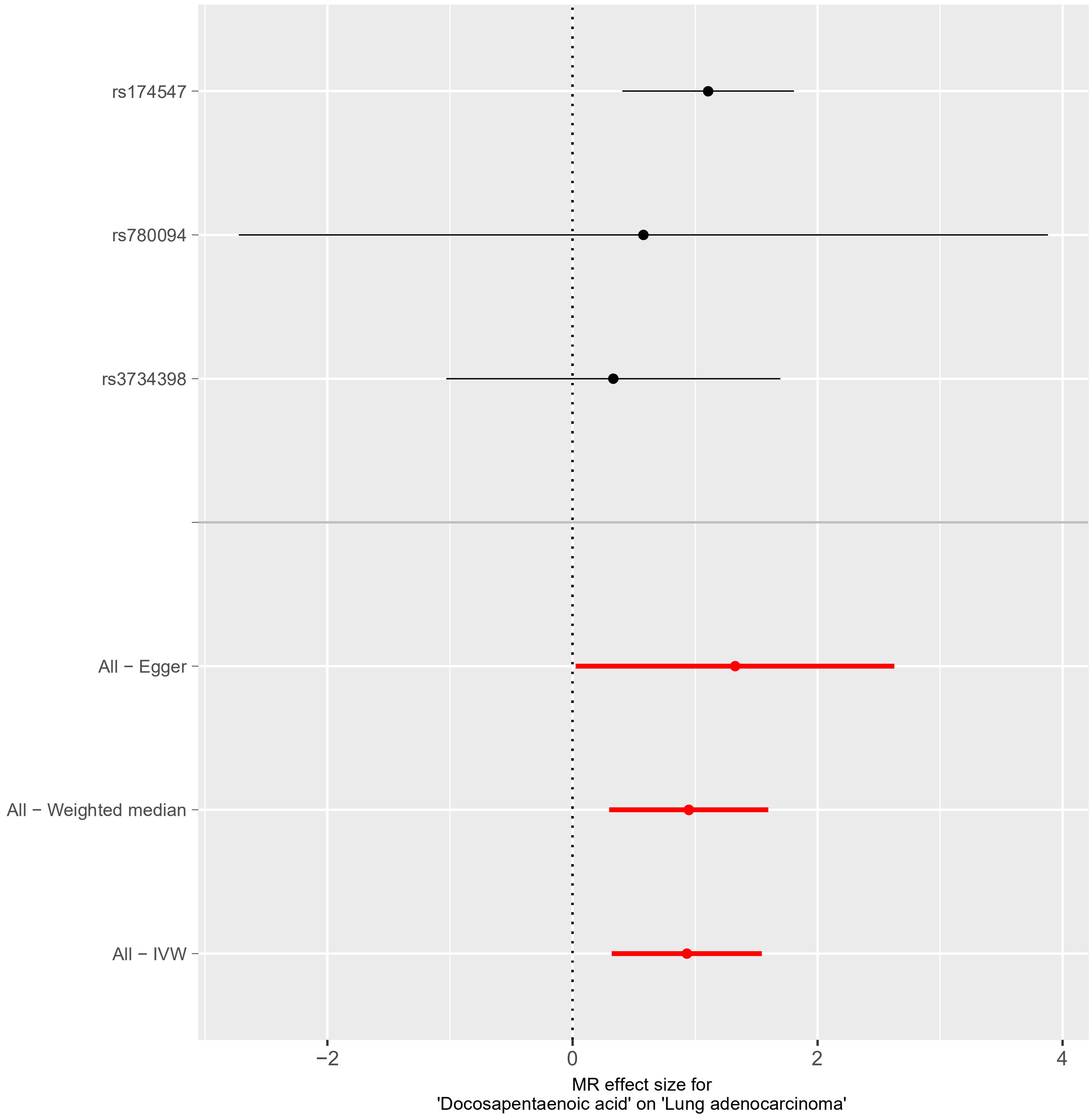

Fig. S1. Forest plot of single nucleotide polymorphisms (SNPs) associated with docosapentaenoic acid (DPA) and their risk of lung adenocarcinoma.

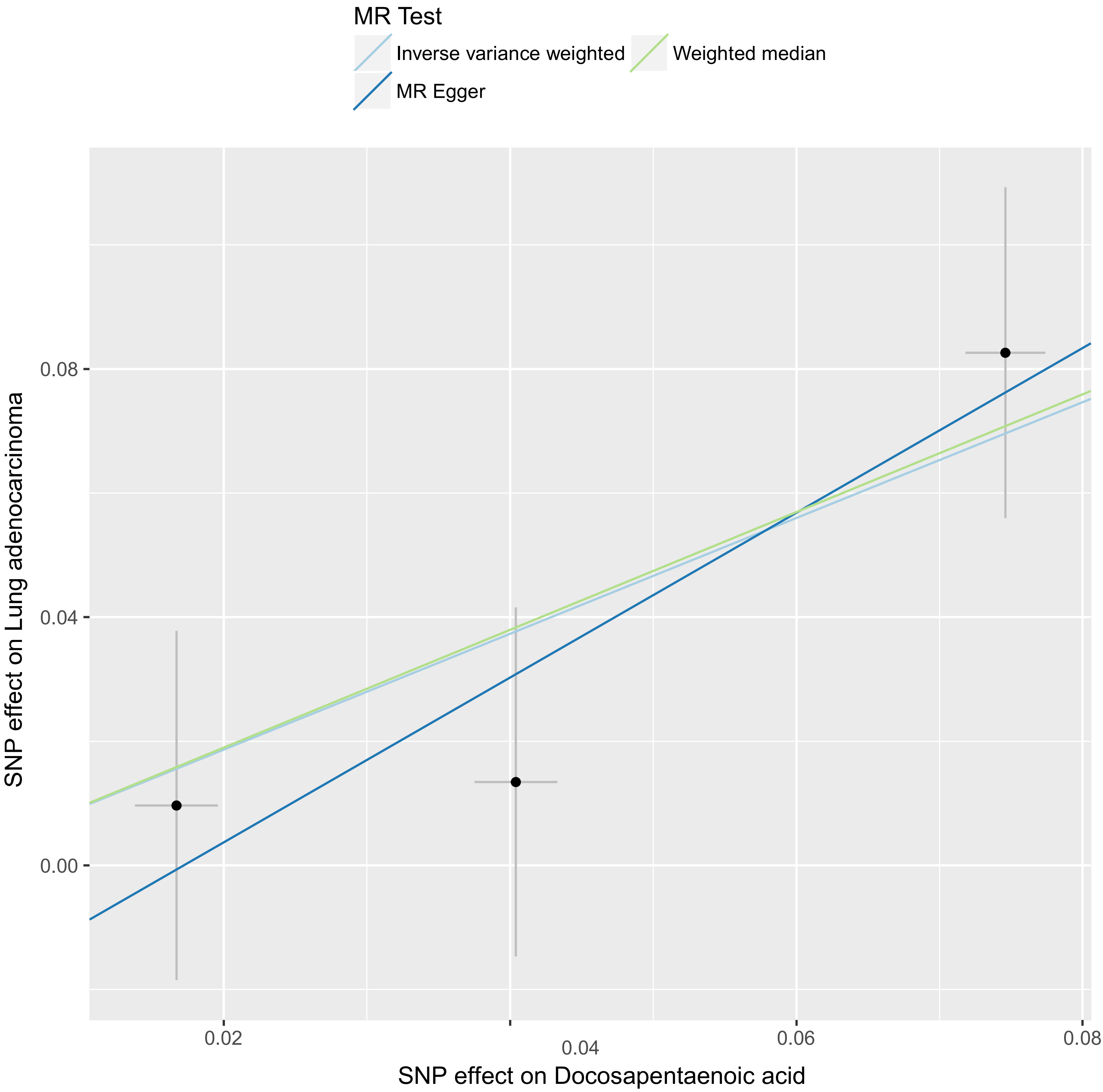

Fig. S2. Scatter plot of SNPs associated with DPA and their risk of lung adenocarcinoma.

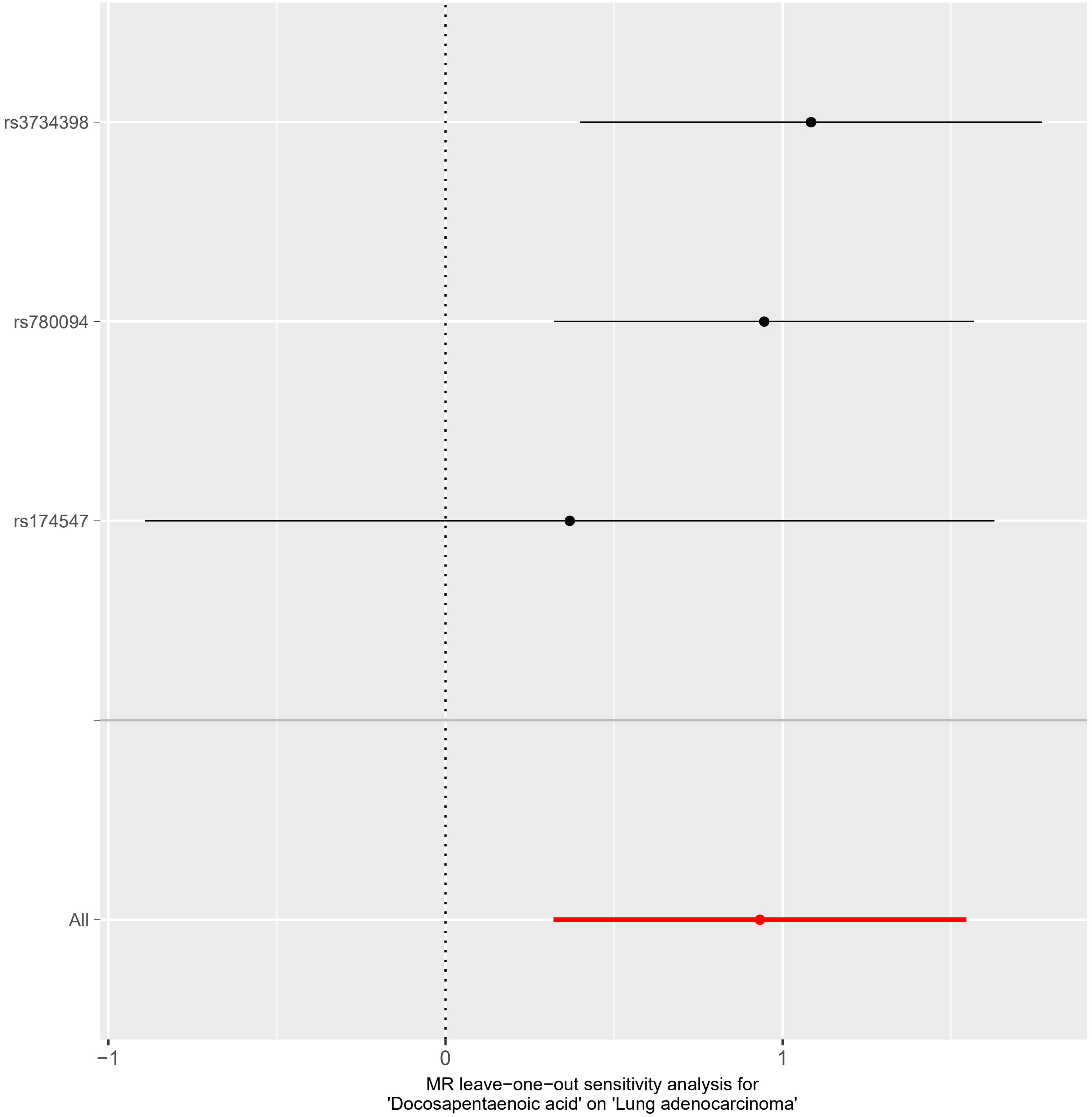

Fig. S3. Leave-one-out of SNPs associated with DPA and their risk of lung adenocarcinoma.

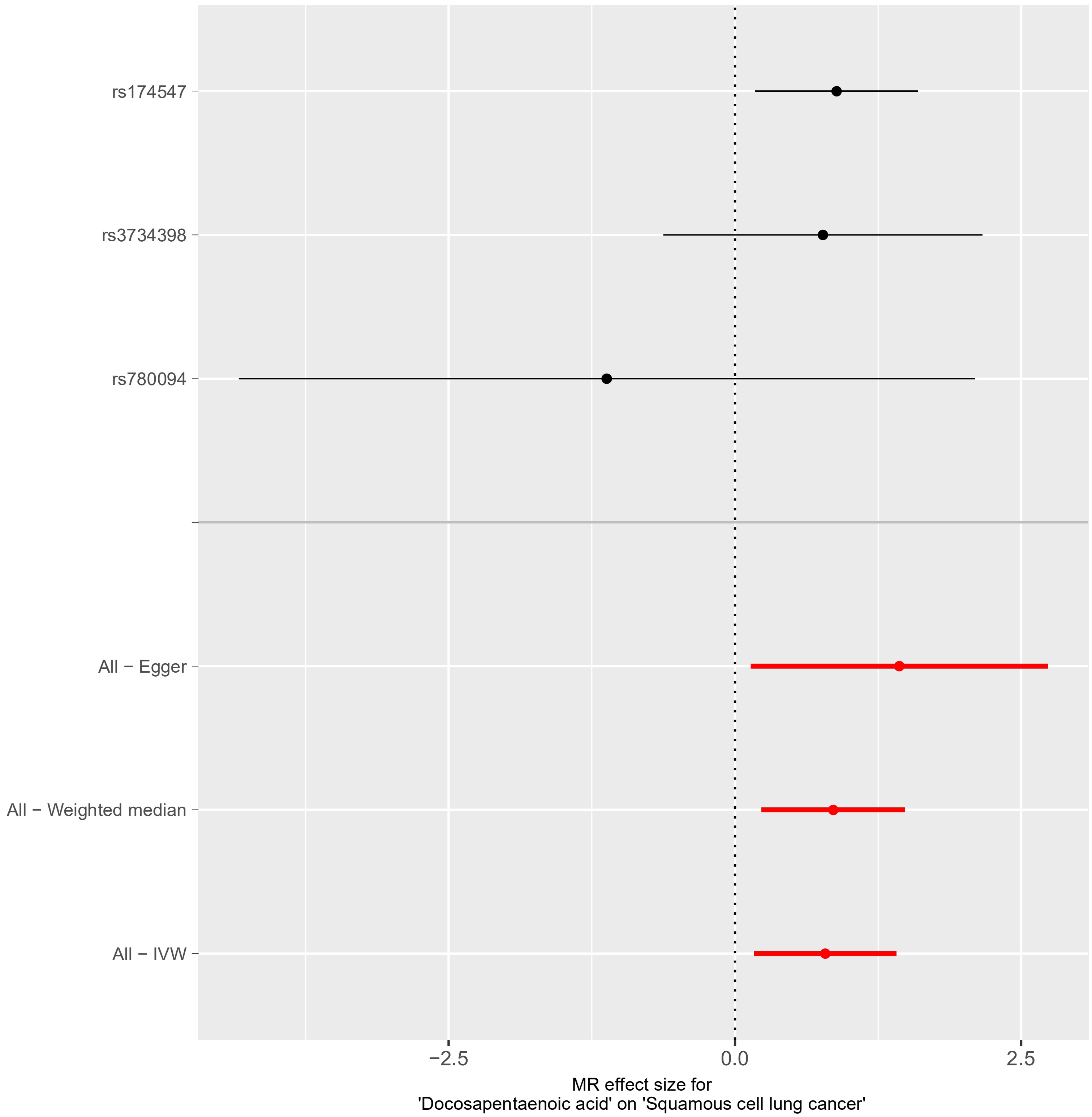

Fig. S4. Forest plot of SNPs associated with DPA and their risk of squamous cell lung cancer.

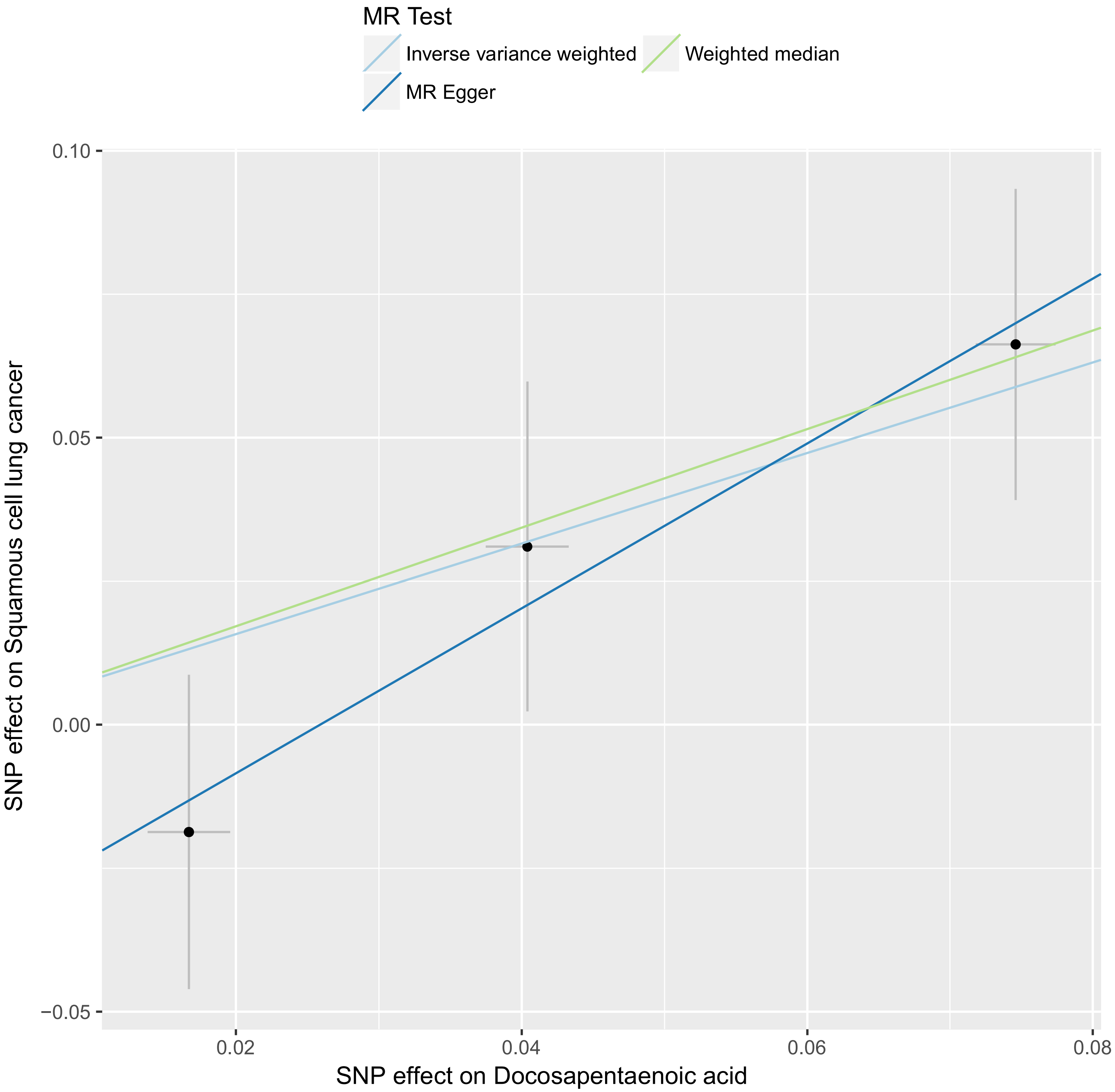

Fig. S5. Scatter plot of SNPs associated with DPA and their risk of squamous cell lung cancer.

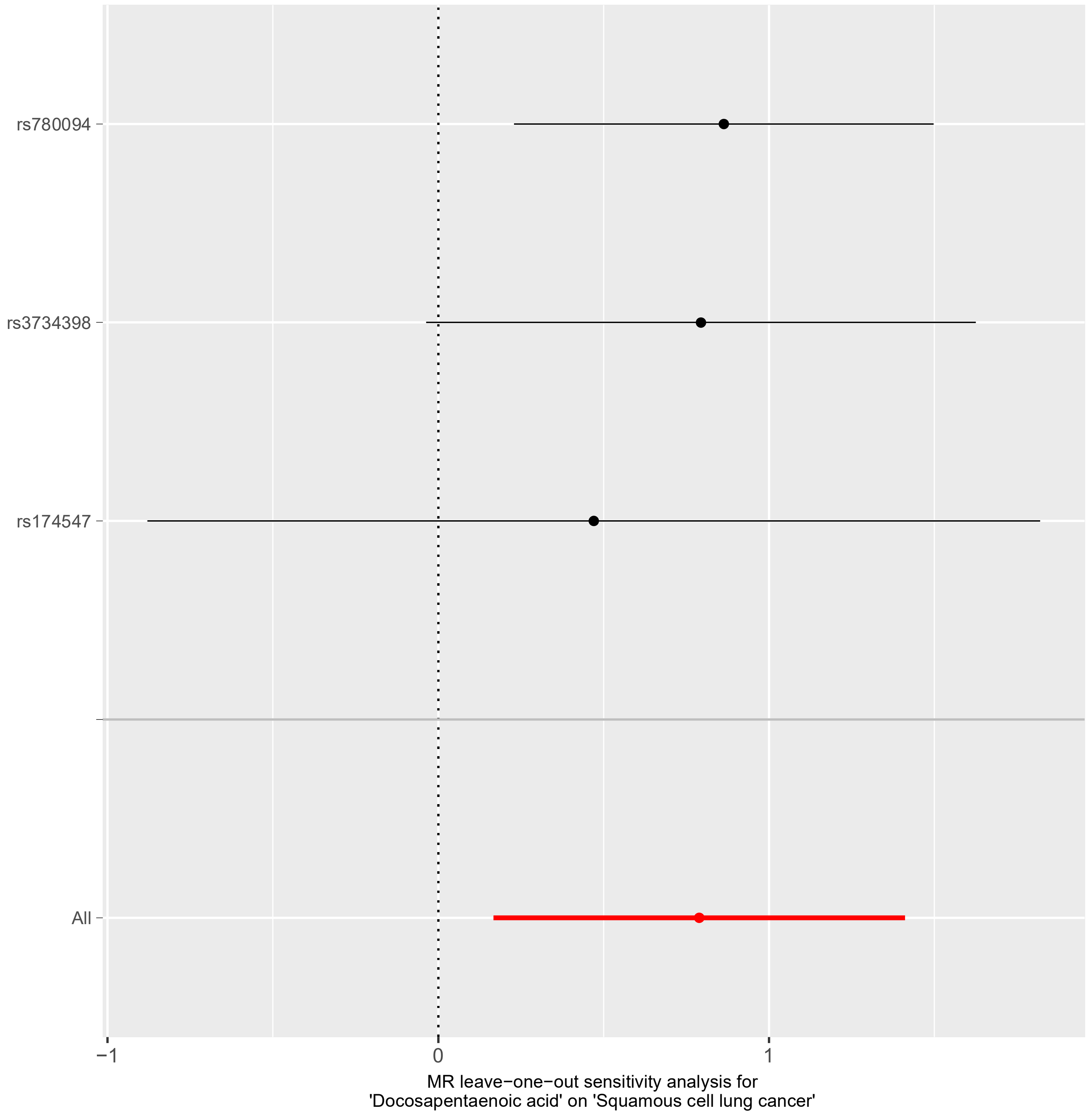

Fig. S6. Leave-one-out of SNPs associated with DPA and their risk of squamous cell lung cancer.

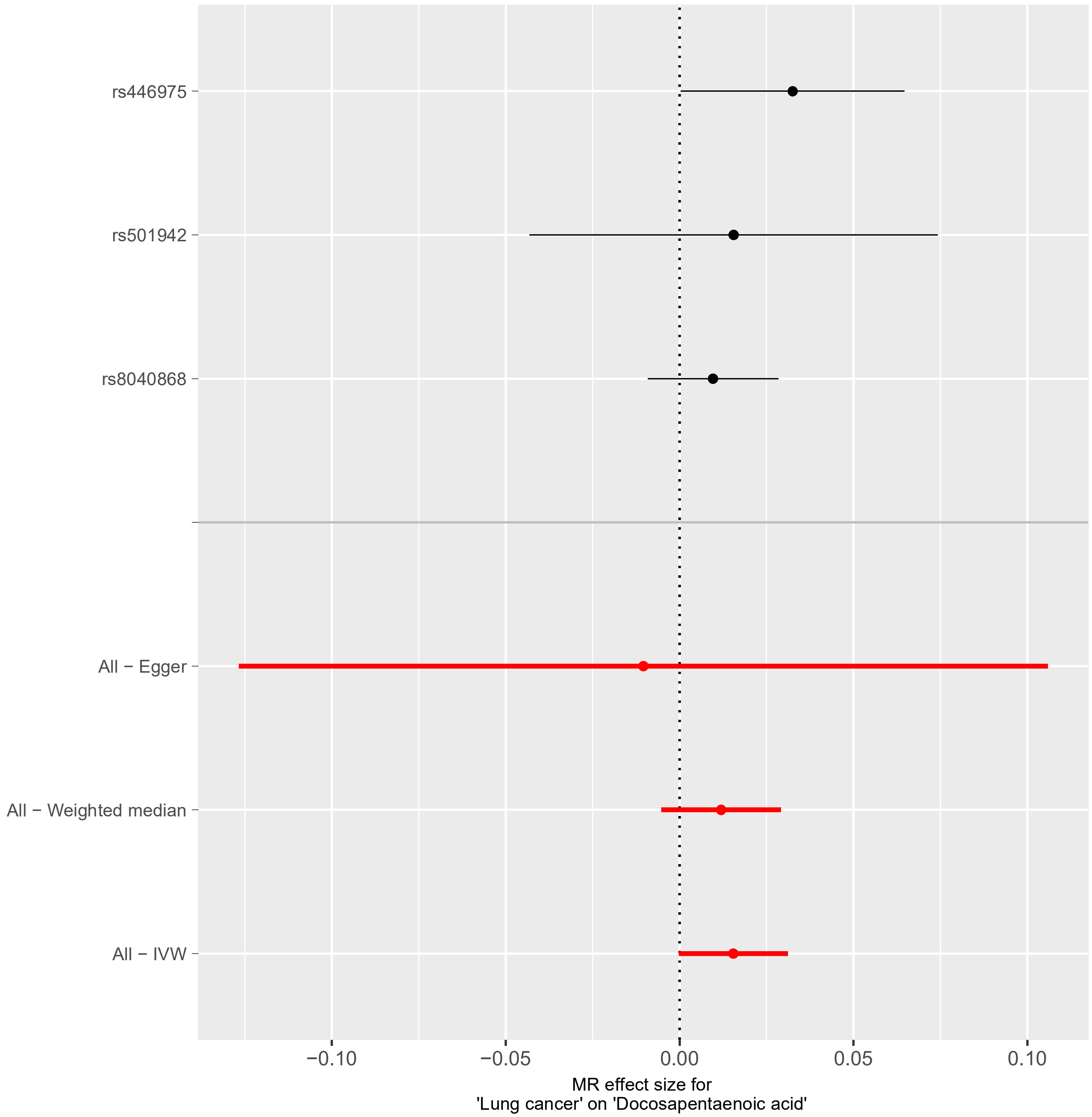

Fig. S7. Forest plot of SNPs associated with lung cancer and their risk of DPA.

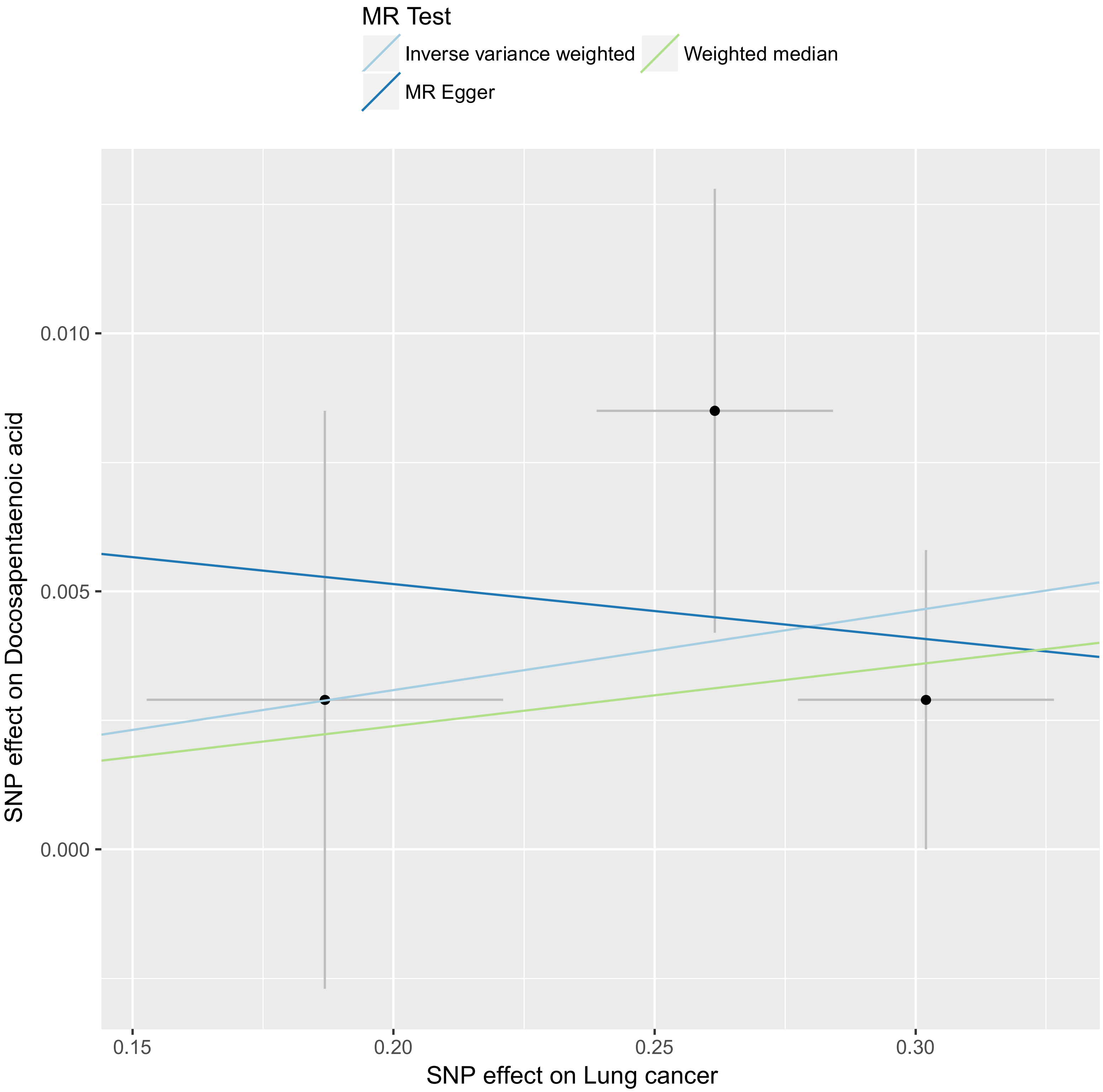

Fig. S8. Scatter plot of SNPs associated with lung cancer and their risk of DPA.

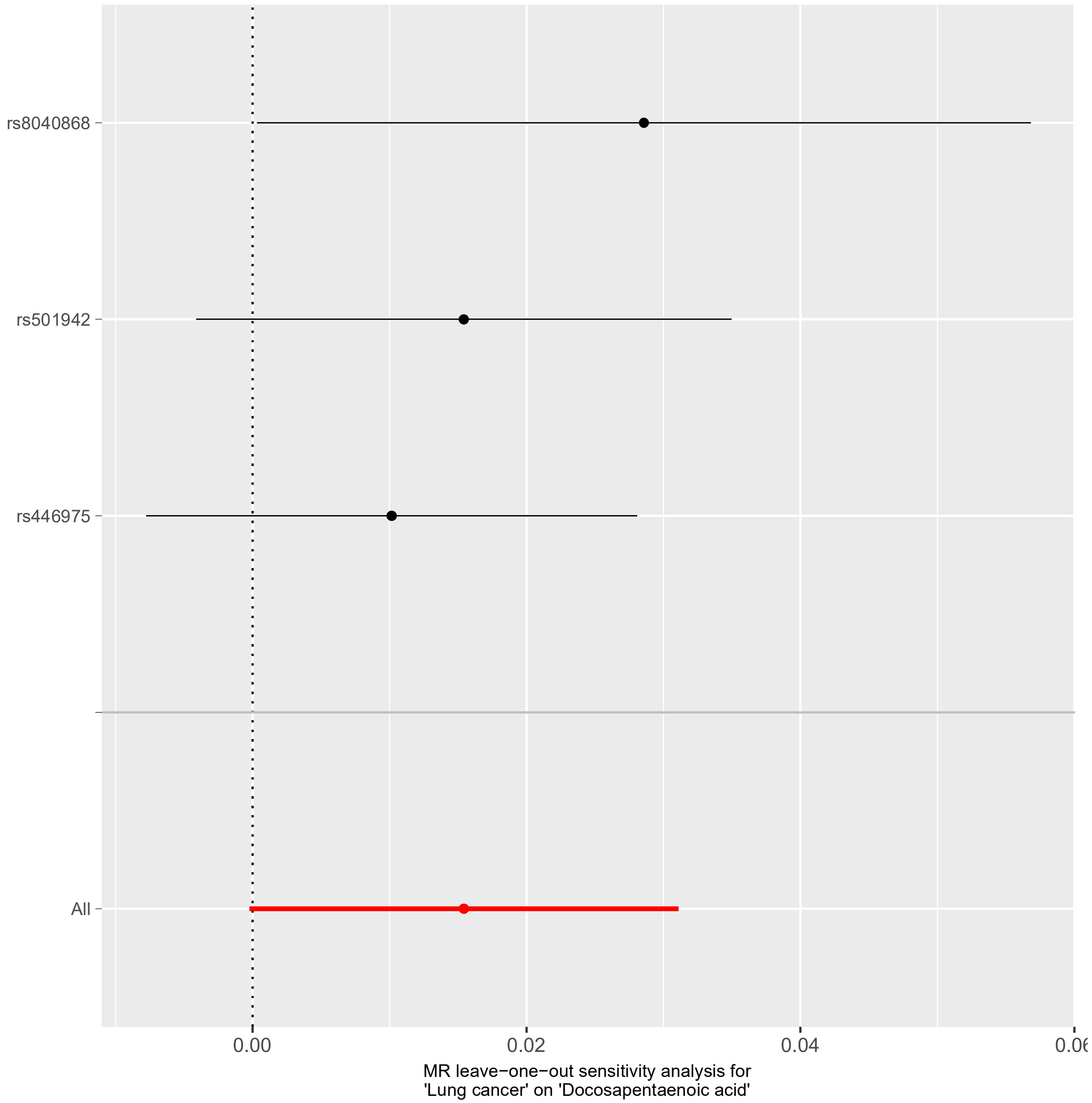

Fig. S9. Leave-one-out of SNPs associated with lung cancer and their risk of DPA.
